# Heritability and Evolutionary Potential Drive Cold Hardiness in the Overwintering *Ophraella communa* Beetles

**DOI:** 10.3389/fphys.2018.00666

**Published:** 2018-06-05

**Authors:** Chenchen Zhao, Fangzhou Ma, Hongsong Chen, Fanghao Wan, Jianying Guo, Zhongshi Zhou

**Affiliations:** ^1^State Key Laboratory for Biology of Plant Diseases and Insect Pests, Institute of Plant Protection, Chinese Academy of Agricultural Sciences, Beijing, China; ^2^Key Laboratory of Biosafety, Ministry of Environmental Protection, Nanjing, China; ^3^Nanjing Institute of Environmental Sciences, Ministry of Environmental Protection, Nanjing, China; ^4^Guangxi Key Laboratory of Biology for Crop Diseases and Insect Pests, Institute of Plant Protection, Guangxi Academy of Agricultural Sciences, Nanning, China

**Keywords:** *Ophraella communa*, heritability, cold hardiness, super cooling point, glycerol content, chill coma recovery time

## Abstract

Chill tolerance plays a crucial role that allows insect species to adapt to cold environments. Two Chinese geographical populations (Laibin and Yangzhou populations) were selected to understand the chill resistance and evolutionary potential in the *Ophraella communa*, a biological control agent of the invasive common ragweed, *Ambrosia artemisiifolia*. Super-cooling point assays, knockdown tests under static low-temperature conditions and determination of glycerol content were studied. ANOVAs indicated significant differences regarding chill coma recovery time, super-cooling point, and glycerol content across populations and sexes. The narrow-sense heritability (*h*^2^) estimates of cold resistance based on a parental half-sibling breeding design ranged from 0.39 to 0.53, and the *h*^2^ value was significantly higher in the Yangzhou population than in the Laibin population. Additive genetic variances were significantly different from zero for cold tolerance. The Yangzhou population of *O. communa* has a strong capability to quickly gain resistance to cold. We conclude that the *O. communa* beetle has a plasticity that can provide cold resistance in the changing climate conditions.

## Introduction

Poikilothermic character is a crucial and widely recognized factor affecting insect development. Fluctuations in environmental temperature can affect the survival, development, and establishment of an insect in the field. Many insect species are currently experiencing habitat destruction due to climate change (Hoffmann et al., 2003), and some are facing previously unencountered low temperatures during the winter months. Cold climates often impede the establishment and persistence of insect populations in temperate regions. Across the long process of evolution, many types of insects have undergone several physiological changes that improved their cold-hardiness (Danks, 1996; Bale, 2002; Chen and Kang, 2002; Wang and Kang, 2005). Cold-hardiness may be a coping strategy that would allow insects to successfully migrate and establish themselves under new environmental conditions (Hoffmann, 2003; Chen et al., 2011; Kellermann et al., 2012a; Findsen et al., 2013). Due to a plastic cold-hardiness, insects can often survive at subzero temperatures during cold winter months (Salt, 1961; Kostal et al., 1998; Overgaard et al., 2007). Many previous studies have suggested that cold tolerance differs significantly across various geographical populations of an insect species mainly due to acclimation to different climatic conditions (Chen and Kang, 2002; Worland et al., 2004; Regniere and Bentz, 2007; Zhou et al., 2011b).

Adaptive phenotypic plasticity and genetic adaptation are the main ways in which natural populations respond to strong environmental stresses. Generally, natural selection can lead to micro-evolutionary changes in some particular traits of wild populations of insects (Merilä et al., 2001; Roff, 2003; Kellermann et al., 2009; Ma et al., 2014). Species distribution is often closely related to the appropriate genetic variation in the key traits which is necessary for adaptation to the changing environmental conditions (Merilä and Crnokrak, 2001). In general, evolutionary change requires that the traits subject to selection will be heritable (Roff, 2003), and thus the key traits for adapting a new environment are often coupled with genetic variation. A rapid evolutionary change in natural populations has provided the evidence for the abundance of genetic variation (Reznick et al., 1997; Hendry and Kinnison, 1999; Kellermann et al., 2009; Ma et al., 2014). Pliability of cold tolerances in insects is also closely associated with the genetic variation to overcome the cold spell in winter months (Gibert et al., 2001; Kimura, 2004; Kellermann et al., 2009; Hoffmann, 2010; Arboleda-Bustos and Segarra, 2011). Nevertheless, the evolution of plasticity, such as cold tolerances for example, under extreme conditions has become increasingly important for natural predators of specific pests increasingly exposed to colder envirenment, and there is an urgent need to excavate empirical information for evolved adaptive phenotypic plasticity.

*Ophraella communa* LeSage (Coleoptera: Chrysomelidae), is native to North America (Futuyma, 1990; Palmer and Goeden, 1991), and it has been considered as an important biological control agent of invasive common ragweed, *Ambrosia artemisiifolia* L. (Asterales: Asteraceae) in China (Chen et al., 2009; Guo et al., 2011; Zhou et al., 2011c, 2014). *A. artemisiifolia* has caused serious economic, ecological, and health problems (Zhou et al., 2011a), and it has extensively invaded and established itself in over 21 provinces in China (Zhou et al., 2011a). *A. artemisiifolia* ages and dies after the end of October, and its seeds in soil germinate and grow new seedlings from late April to early June next year. *O. communa* adults often hibernate in soil from the end of October to early May or June of the following year. In general, air temperatures differ significantly with latitudes. The hibernating *O. communa* adults from different latitudes often need to overcome different low temperatures in the cold winter months. Our previous studies have demonstrated that hibernating *O. communa* use freeze avoidance to survive winter cold and geographically separated populations of *O. communa* are diverging with respect to baseline cold hardiness because of the differing severity of low temperatures experienced during the coldest winter season in each locality (Zhou et al., 2011a). The Yangzhou population may be one of the most northern populations of this species which ranges from Laibin, Guangxi Province, north to the border of Yangzhou City, Jiangsu Province. Moreover, significant differences were observed in cold hardiness across different seasonal populations of *O. communa* have been reported (Zhou et al., 2011b). This indicates the cold-hardiness plasticity of *O. communa* plays an important role in adapting to seasonal and geographic changes in air temperatures.

As *A. artemisiifolia* gradually expands northward, *O. communa* encounters a colder environment, and new selection pressures may result in genetic variation within the species and may even result in the rapid adaptive microevolution of chill tolerance traits. Short-term evolutionary potential depends on the intra-specific additive genetic variance. The additive variance is often measured as heritability, the fraction of the total phenotypic variance that is additive. In this way, heritability is a common measure of evolutionary potential. In general, the proportion of intra-population phenotypic variance (*V*_P_) due to additive genetic variation, termed narrow-sense heritability (*h*_2_), may be the most fundamental aspect of a trait’s genetic architecture, and has evolutionary significance (Lynch and Walsh, 1998; Visscher et al., 2008; Hoffmann and Sgro, 2011). In this study, coefficient of additive genetic variance (*CV*_A_) served as a measure of evolvability, because its numerical value has a more direct interpretation as the expected relative change in a trait under a unit strength of selection (Houle, 1992; Sgro and Hoffmann, 1998; Hansen et al., 2003). Few empirical studies have clearly demonstrated how the transgenerational effects contribute to insects’ adaptability to cold conditions. Previous studies in *Drosophila melanogaster* and *Bemisia tabaci* have produced evidence of adaptive cross-generation plasticity, which has suggested that variance in evaluability is greater when insects are exposed to a changing temperature stress (Cui et al., 2008; Mitchell and Hoffmann, 2010; Ma et al., 2014). Species with adequate genetic variability are often able to make rapid evolutionary adjustments in nature. In this way, temperature resistance plasticity of insects may be heritable (Ma et al., 2014). Therefore, understanding the evolutionary potential in cold resistance plasticity can help predict the future responses of *O. communa* to climate change.

In the present study, we hypothesize that the cold-hardiness plasticity of overwintering *O. communa* adults should be heritable in geographically separated cold climates in the winter months. Two geographical populations (Laibin and Yangzhou) were selected to estimate specific parameters crucial to cold tolerance, including super cooling point (Lee, 1989; Chen and Kang, 2002; Andreadis and Athanassiou, 2017), glycerol content (Krunic and Salt, 1971; Minder et al., 1984), and chill coma recovery time (Findsen et al., 2013) of the adults. The purpose of this study was to compare the evolutionary potential of the baseline cold hardiness of hibernating *O. communa* populations from two different geographical regions, and so provide insight into the potential of this beetle to range further into China. To this end, we used laboratory measurements to compare the heritability of cold hardiness in overwintering *O. communa* adults from the two geographical populations. Based on estimation of the heritability of cold hardiness, we will explain how *O. communa* establishes its populations successfully to survive cool climates during winter months in different latitude localities of China.

## Materials and Methods

### Host Plants and Insects

The beetle *Ophraella communa* LeSage can survive throughout the year on the common ragweed (*Ambrosia artemisiifolia* L.) from May to November in the open fields and hibernate in the soil during the winter months.

The overwintering *O. communa* adults were collected from naturally grown ragweed, in the fields of Laibin (Guangxi Zhuang Autonomous Region, South China) and Yangzhou (Jiangsu Province, East China) when they emerged from the soils in the spring 2014. The adults were maintained (two geographical populations were reared separately) in the laboratory at a temperature of 27 ± 1°C, a relative humidity of 70 ± 5% RH, and a light dark photoperiod of 14:10 h. The beetles were bred gregariously in insect rearing cages (40 cm × 60 cm). The cages contained ragweed plants for eating, climbing, molting, and hiding. The moisture levels (15.5–18.5%) were maintained along with sterilized soil to facilitate ragweed growth.

**FIGURE 1:**
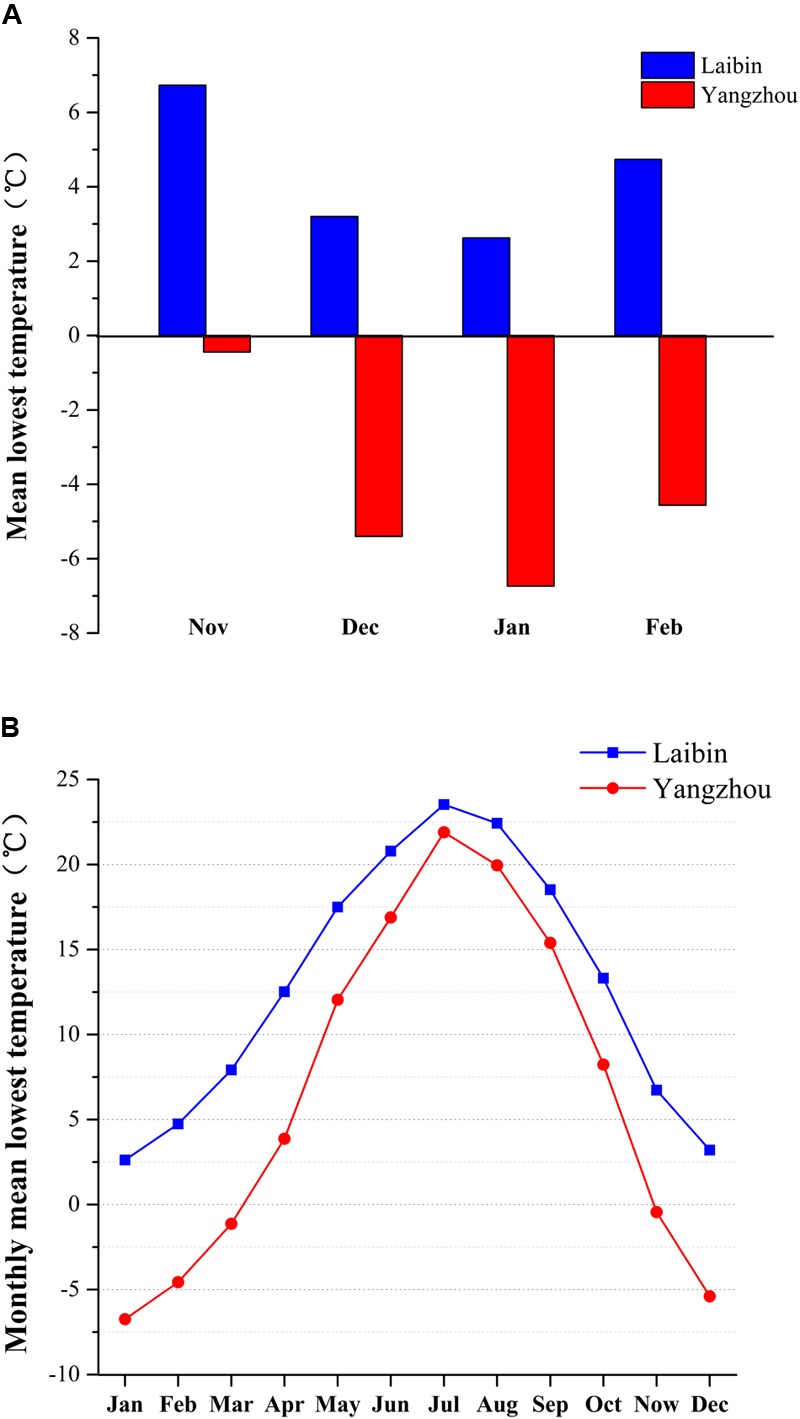
Mean lowest temperature, monthly mean low temperature, and monthly mean temperatures in Laibin and Yangzhou, China from 2007 to 2014. Data were obtained from the official website of China Meteorological Data Sharing Service System at http://data.cma.cn. The weather station providing these data is located in Laibin, Guangxi (23°43′7.47″N, 109°24′54.10″E) and Yangzhou, Jiangsu (32°16′12.53″N, 119°10′31.99″E). **(A)** The average monthly minimum temperatures in the winter months from 2007 to 2014. **(B)** The averages from daily minimum temperature in each month from 2007 to 2014.

Laibin belongs to subtropical monsoon climate and its location is 23°43′7.47″N, 109°24′54.10″E, where the average lowest temperatures in November, December, January, and February were 6.7°C, 3.2°C, 2.6°C, and 4.7°C, respectively, and the average low temperatures in November, December, January, and February were 14.1°C, 10.3°C, 8.2°C, and 12.1°C, respectively. Yangzhou also belongs to subtropical monsoon climate and its location is 32°16′12.53″N, 119°10′31.99″E, where the average lowest temperatures in November, December, January, and February were -0.4°C, -5.4°C, -6.7°C, and -4.6°C, respectively, and the average low temperatures in November, December, January, and February were 7.7°C, 1.7°C, -0.5°C, and 2.1°C, respectively. Although Laibin and Yangzhou both belong to subtropical monsoon climate, Laibin have higher temperature all year-round, especially in winter (**Figure 1**). The data were provided by the China meteorological data service center (CMDC)^1^.

### Experimental Design and Data Collection

After a generation, male and female beetles’ generation 1 (G1) were transferred to separate plastic boxes (24 cm × 16 cm × 9 cm) covered with organdy mesh fabric. These were placed in a temperature-regulated laboratory within 24 h, and then maintained for 2 days on common ragweed plants in insect rearing cages. The two geographical populations were reared for one generation to ensure a large, effective population size.

### Paternal Half-Sibling Breeding Design

When the adult beetles of generation 2 (G2) were 3 days old (sexual maturity), we randomly assigned one male (sire) to mate with five virgin females (dams) in turn, in a transparent plastic box containing a glass vial (3 cm × 8 cm) with a ragweed branch from each geographical population (*N* = 30 for one population). The ragweed branch was for mating, oviposition, climbing, molting, and hiding. After mating was complete, we gently removed each female to a new “house,” numbered them by mating pair, then placed another female in the old “house.” After all pairs had finished mating, we checked females’ oviposition daily. When the female laid her eggs, was removed from the vial by aspiration immediately, left the eggs behind. Then we observed the eggs until they hatched.

When their offspring, generation 3 (G3) emerged, 10 females of each combination were collected, reared on ragweed plants, mated with males, and scored for cold tolerance traits (two females for cold stress, two females for testing of super-cooling point and glycerol content separately) within 3 h of eclosion. The breeding experiment was performed in the laboratory at 27°C, at humidity of 65–75% RH under a photoperiod of 14L: 10D cycle with light coming on at 08:00.

### Chill Coma Recovery Time-T*_RC_*

To compare the cold resistance between the populations and sexes, 400 individuals were randomly selected from each mass bred population and scored for knockdown resistance (100 females and 100 males per cold stress experiment) on the third day after eclosion.

Five adults were randomly selected from every replication, the centrifuge tubes were paced (one adult/tube) in temperature controller (-10 ± 0.1°C). The centrifuge tubes were removed, immediately rubbed dry, and placed on a piece of paper, where they were allowed to recover at room temperature (25°C) without any mechanical stimuli. Then we started a timer observed their activity, noted the recovery time, measured as period from placement on the paper to the beetle reaching an upright position.

### Super-Cooling Point

We randomly selected 150 males and 150 females from both geographical populations, a thermocouple was used to test the super-cooling point, we cut one 2 mm aperture of the pipet tips, then placed the probe of thermo detector in said pipet tips. Connect the probe with the insect body, then put it in a low temperature refrigerator (-20°C and -30°C). Insects obtain marked cooling at an average rate of about 0.5°C/min, when the body fluids starts turning to ice, latent heat is released within the insect, producing a large bending curve in the recorder, distinguished the super-cooling point (when the curve rose to a certain value, and began to decline; we recorded the freezing point) numbered and recorded the data.

### Determination of Glycerol Content

Glycerol contents were estimated from 100 randomly selected males and 100 randomly selected females from both geographical populations (400 specimens in total). Experimental procedures were conducted according to the kit instructions (provided by Nanjing Jiancheng Bioengineering Institute) and data were recorded.

### Statistical Analysis

Data were analyzed using mixed modeling within a restricted maximum likelihood framework implemented in Proc Mixed in SAS (version 9.2. SAS Institute Inc., Cary, NC, United States) with following the model:

(1) $$y\;=\alpha +XB+Z_{s}d_{s}+Z_{d}d_{d}+\;e$$

Here the constant (α) was ensemble average of each protocol. A previous study suggested that observer error may affect the estimation of variance components for thermal tolerance traits (Castaneda et al., 2012). All three protocols, (knockdown test, super-cooling point assays, and determination of glycerol content), were performed by a single person. We here considered protocol run (B) as a fixed effect, and three random effects were fit: sire (d_s_), dam nested within sire (d_d_), and the residual (e.).

The total phenotypic variance ($\sigma_{P}^{2}$) for the breeding design for estimating genetic parameters was represented as follows:

$$\sigma_{P}^{2}=\sigma_{S}^{2}+\sigma_{D}^{2}+\sigma_{W}^{2}(2)$$

Here $\sigma_{S}^{2}$, $\sigma_{D}^{2}$, and $\sigma_{W}^{2}$, are the sire, dam, and intra-group level variance components, respectively. Because we used a half-sib breeding design, the sire variance, $\sigma_{S}^{2}$, is one-fourth of the additive genetic variance (*V*_A_) (McGuigan and Blows, 2010). Thus, to estimate *V*_A_, we multiplied the sire variance by four.

The additive genetic variance for each trait was first estimated using a univariate model. Log likelihood ratio tests were performed, where the final model for each trait was compared to a model in which _$\sigma_{S}^{2}$_ was set to zero to determine whether the levels of additive genetic variance for each trait were significantly different from zero (de Assis et al., 2010; McGuigan and Blows, 2010; Simonsen and Stinchcombe, 2010). The phenotypic variance (*V*_P_) in knockdown resistance was computed using all known relationships among individuals. We then estimated the narrow-sense heritability for both traits. Narrow-sense heritability for each trait was estimated as the additive genetic variance (*V*_A_) divided by the total phenotypic variance (*V*_P_) (Frankham, 1996). We conducted Student’s *t*-tests to determine whether the variance components and heritability estimates differed significantly. Estimates of evolvability, *I*_A_, and the additive genetic coefficient of variance,

$$I_{A}=V_{A}/\bar{X^{2}}$$

where $\overset{-}{X}$ is the trait mean (Hansen et al., 2011), were also computed for both traits.

For the population and sexual comparisons (G3), two-way ANOVA was performed to evaluate the differences in the indexes, followed by the Tukey multiple comparison test. The population (Laibin and Yangzhou) and sex (female and male) were used as the two factors. *P* < 0.05 was considered statistically significant.

## Results

### Differences in Cold Resistance Between the Populations and Sexes

**FIGURE 2:**
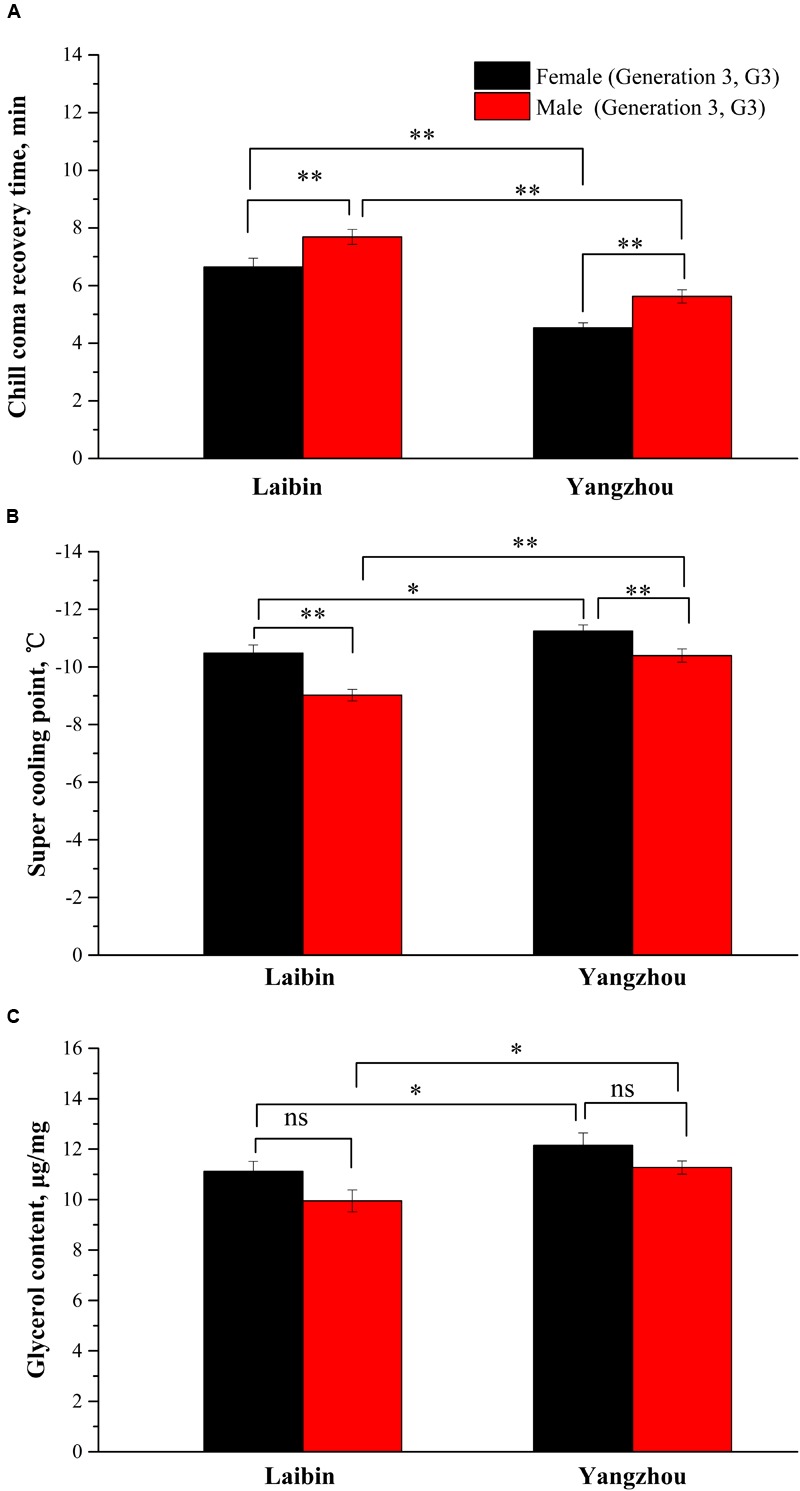
Index of cold resistance between sexes. **(A)** Chill coma recovery time (min). **(B)** Super-cooling point (°C). **(C)** Glycerol content (μg/mg) for the Laibin (G3) and Yangzhou population (G3) of *Ophraella communa* beetles (mean ± SE). ^∗^*P* < 0.05, ^∗∗^*P* < 0.01.

**Figure 2** showed the mean value of *T_rc_*, SCP, and GC in two populations of *O. communa* beetles (G3). An ANOVA indicated significant differences between the two populations (*F*_3,178_= 74.99, *P* < 0.0001) and between males and females (*F*_3,178_= 19.55, *P* < 0.0001) in *T_rc_* (**Figure 2A**). Similar results were observed for SCP of population (*F*_3,199_ = 20.77, *P* < 0.0001) and sex (*F*_3,199_ = 23.13, *P* < 0.0001), and GC of population (*F*_3,397_ = 10.41, *P* = 0.0014) and sex (*F*_3,397_ = 9.01, *P* = 0.0029). Generally females showed markedly more cold resistance than males.

### Heritability and Genetic Variance Components

As shown in **Table 1**, the estimates of genetic variance differed statistically significantly between the two populations, Narrow-sense heritability estimates were significantly different for the chill coma recovery times of both the Laibin and Yangzhou populations, at values of 0.39 ± 0.01^∗^ and 0.53 ± 0.01^∗^, respectively. Significant levels of additive genetic variance were detected in chill coma recovery time (2.15 ± 0.01) of the Laibin population and in chill coma recovery time (5.44 ± 0.02) and super-cooling point (1.67 ± 0.22) of the Yangzhou population. For chill coma recovery time, the narrow-sense heritability estimates were significant for both the Laibin (0.39 ± 0.01^∗^) and Yangzhou populations (0.53 ± 0.01^∗^). The coefficient of environmental variation was slightly larger (*CV*_E_ of glycerol content, 30.34 > 27.37) for the Laibin population and that of and additive genetic variation was slightly smaller (*CV*_A_ of chill coma recovery time, 0.539 < 1.111; *CV*_A_ of super-cooling point, 0.181 < 0.393; *CV*_A_ of glycerol content, 0.231 < 0.250).

**Table 1:** Narrow-sense heritability (*h*^2^), variance, and coefficient of variation components for chill coma recovery time (*T_rc_*), super-cooling point (SCP), and glycerol content (GC) (Laibin and Yangzhou populations) of *Ophraella communa beetles*.

Mean/Variance^∗^	Laibin population (G3)			Yangzhou population (G3)		
	*T_rc_*, min	SCP (–)	GC	*T_rc_*, min	SCP (–)	GC
*Mean* ± SE	7.39 ± 0.65	9.75 ± 0.93	10.53 ± 0.40	4.41 ± 0.24	10.82 ± 1.27	11.81 ± 0.25
*V*_A_ ± S.E.	2.15 ± 0.01^1^	0.32 ± 0.13	0.56 ± 0.09	5.44 ± 0.02^1^	1.67 ± 0.22^1^	0.74 ± 0.01
*V*_E_ ± S.E.	5.56 ± 0.44	2.25 ± 0.51	10.77 ± 0.55	10.21 ± 0.48	9.94 ± 0.80	11.19 ± 0.60
*V*_P_ ± S.E.	1.59 ± 0.02	2.70 ± 0.05	3.15 ± 0.07	2.06 ± 0.06	4.46 ± 0.01	2.77 ± 0.03
*h*^2^ ± S.E.	0.39 ± 0.01^∗^	0.14 ± 0.08	0.05 ± 0.01	0.53 ± 0.01^∗^	0.17 ± 0.02	0.07 ± 0.01
*I*_A_	0.291	0.033	0.053	1.234	0.154	0.063
*CV*_A_	0.539	0.181	0.231	1.111	0.393	0.250
*CV*_E_	24.99	14.26	30.34	49.51	26.58	27.37
*N*	110	110	110	124	124	124

*Additive genetic variance (*V*_*A*_), environmental variance (*V*_*E*_), phenotypic variance (*V*_*P*_), narrow-sense heritability (*h*^*2*^), evolvability (*I*_*A*_ × 100), coefficient of additive genetic variance (*CV*_*A*_), and coefficient of environmental variance (*CV*_*E*_) for chill coma recovery time, super-cooling point and glycerol content. *N* = sample size. ^*1*^*P* < 0.05 for log likelihood ratio test of significant differences from zero; ^∗^*P* < 0.05.*

## Discussion

Different populations react differently to cold stress. What one population finds very stressful another may not (Overgaard et al., 2011; Hoffmann, 2013). Temperature has an important influence on insect population distribution, life history, behavior, and species abundance (Dahlgaard et al., 2001; Kimura, 2004; Hoffmann, 2010). For many insects, chill tolerance is crucial to the ability to persist in cold environments (Findsen et al., 2013). Cold resistance is associated with the distributions of many insect species, particularly in temperate regions. Insects have low levels of resistance. *A. artemisiifolia* has become distributed across over 21 provinces from Southern to Northern China, covering a range of different climatic temperatures (Guo et al., 2011), its seeds grow new seedlings from late April to early June next year (Teshler et al., 2001). *O. communa* can still survive the cold winter months in the field (Zhou et al., 2011b). In the research studies conducted, we found that the hibernation survival range of beetle population in Langfang City, Hebei Province (39°30′38″N, 116°36′2″E) (Zhou, unpublished data), is still smaller than the range of ragweed. Previous studies have suggested there may be significantly differences in cold hardiness among different seasonal and geographic populations of *O. communa* (Zhou et al., 2011a,b), and it is important to determine whether cold tolerance can be inherited by different geographic populations.

In our study, we have further documented genetic differences in cold tolerance between populations of *O. communa*. The Yangzhou population was found to be more cold-tolerant than in the Laibin population. This indicates a significant difference in the adaptive strategies to cold climates between the Laibin and Yangzhou populations of *O. communa*. As shown, the Yangzhou population can survive in a colder climatic environment than the Laibin population. In general, adapting and shifting their geographical distributions rendered some species better adapted to their environment (Kirkpatrick and Peischl, 2012). Previous studies have suggested that conspecific populations of the fruit fly *Drosophila melanogaster* from different environments may vary substantially in stress resistance because of acclimation to local conditions (Hoffmann et al., 2005). This is particularly evident when exploring the relationship between cold tolerance and minimal environmental temperature, such that strong correlations exist between cold tolerance and environmental distribution (Chen et al., 2011; Kellermann et al., 2012b).

Given adequate genetic variability, insect species are often able to make rapid evolutionary adjustments. Under pressure of the global climate warming, the majority of studies on insect adaptation have focused on the heat resistance of poikilotherms. Estimates of heritability in *D. melanogaster* based on knockdown time experiments at 39°C suggest values ranging from 0.03 to 0.28 (Huey et al., 1992; McColl et al., 1996), whereas experiments at 38°C suggest values ranging from 0.14 to 0.22 (Mitchell and Hoffmann, 2010). These estimates might not be directly comparable to those obtained here, which ranged from 0.47 to 0.51 at 45°C, due to the difference in the temperature used to assess the trait and to the different species examined. Nevertheless, the values obtained here showed that a substantial proportion of the total phenotypic variation in heat resistance was caused by additive genetic variation. Few studies have examined the genetic variance of cold resistance (Huey et al., 1992; Charo-Karisa et al., 2005; Kellermann et al., 2009). Estimates of heritability on recovery from a chill coma at 0°C suggest values ranging from 0.01 to 0.38 (Charo-Karisa et al., 2005). Our estimates of heritability for *O. communa* based on chill coma recovery time at -10°C (0.39–0.53) also indicated that a substantial proportion of the total phenotypic variation was caused by additive genetic variation.

Insects typically need to overcome cold conditions in order to become established in a given habitat (Bale, 1996). Cold acclimation, heritability and evolutionary potential could all drive cold hardiness (Sinclair et al., 2003). Cold acclimation was found to have enormous benefits at low temperatures, such that only cold-acclimated flies were able to find resource when they were released into cold environments (Kristensen et al., 2008). Because the insects that prey on pest species tend to follow insect pests into new areas, cold resistance is an important part of their arsenal. *A. artemisiifolia* has spread from south to north in China, so *O. communa* needs to adapt more colder winter climates if it is to suppress the population of *A. artemisiifolia* in colder areas in the northern subtropics or even in temperate regions. Based on the results of our study, we found *O. communa* populations to have considerable evolutionary potential with respect to cold tolerance in colder areas, and its heritability and evolutionary potential drive cold hardiness. This indicates that the beetle has a good plasticity of cold tolerance in nature, so it may be able to overcome the cold climate in Northern China whether the beetle experiences further artificial or natural cold acclimation.

## Author Contributions

ZZ conceived and designed the work, and also edited the manuscript. CZ and FM performed the experiments and wrote the manuscript. HC and JG helped with the theoretical analysis. FW helped to revise the manuscript.

## Conflict of Interest Statement

The authors declare that the research was conducted in the absence of any commercial or financial relationships that could be construed as a potential conflict of interest.

## Footnotes

1 http://data.cma.cn/
